# Nipah virus attachment glycoprotein ectodomain delivered by type 5 adenovirus vector elicits broad immune response against NiV and HeV

**DOI:** 10.3389/fcimb.2023.1180344

**Published:** 2023-07-27

**Authors:** Xiaoyan Huang, Yaohui Li, Ruihua Li, Shaoyan Wang, Lu Yang, Shuyi Wang, Ying Yin, Xiaodong Zai, Jun Zhang, Junjie Xu

**Affiliations:** ^1^ Laboratory of Vaccine and Antibody Engineering, Beijing Institute of Biotechnology, Beijing, China; ^2^ College of Life Science and Technology, Beijing University of Chemical Technology, Beijing, China

**Keywords:** Nipah virus, Hendra virus, vaccine, attachment glycoprotein, adenovirus vector, T-cell epitope

## Abstract

Nipah virus (NiV) and Hendra virus (HeV) are newly emerging dangerous zoonotic pathogens of the *Henipavirus* genus of the *Paramyxoviridae* family. NiV and HeV (HNVs) which are transmitted by bats cause acute respiratory disease and fatal encephalitis in humans. To date, as there is a lack of antiviral drugs or effective antiviral therapies, the development of vaccines against those two viruses is of primary importance, and the immunogen design is crucial to the success of vaccines. In this study, the full-length protein (G), the ectodomain (Ge) and the head domain (Gs) of NiV attachment glycoprotein were delivered by the replication-defective type 5 adenovirus vector (Ad5) respectively, and the recombinant Ad5-NiV vaccine candidates (Ad5-NiVG, Ad5-NiVGe and Ad5-NiVGs) were constructed and their immunogenicity were evaluated in mice. The results showed that all the vaccine candidates stimulated specific humoral and cellular immune responses efficiently and rapidly against both NiV and HeV, and the Ad5-NiVGe elicited the strongest immune responses after a single-dose immunization. Furthermore, the potent conserved T-cell epitope DTLYFPAVGFL shared by NiV and HeV was identified in the study, which may provide valid information on the mechanism of HNVs-specific cellular immunity. In summary, this study demonstrates that the Ad5-NiVGe could be a potent vaccine candidate against HNVs by inducing robust humoral and cellular immune responses.

## 1 Introduction

Nipah virus (NiV) is a single-stranded, negative-stranded RNA virus that belongs to the *Henipavirus* genus of the *Paramyxoviridae* family, along with the closely related Hendra virus (HeV). Both NiV and HeV (henipaviruses, HNVs) are classified as biosafety level 4 (BSL-4) pathogens (Williamson and Torres-Velez, 2010). *Pteropus bats* (flying foxes) have been identified as the natural host of these two viruses (Field et al., 2001; Chua, 2003). According to genetic differences, NiV can be subdivided into two strains, namely the Malaysia strain (NiV-My) and the Bangladesh strain (NiV-Bd). NiV-My and NiV-Bd share around 92% nucleotide identity, indicating limited genomic variation, whereas their translated proteins encoding nucleocapsid (N), phosphoprotein (P), matrix (M), fusion glycoprotein (F), attachment glycoprotein (G) and long polymerase (L) show similarity greater than 92% (Wacharapluesadee and Hemachudha, 2007).

NiV can transmit directly from *Pteropus bats* to humans, causing respiratory and neurological diseases with high pathogenicity and mortality. The first NiV outbreak was among pig farmers in Malaysia in 1998, and pigs were shown to be the intermediate hosts (Lam and Chua, 2002). The Bangladesh strain NiV outbreak was first observed in West Bengal in 2001 (Chadha et al., 2006). Since then, NiV outbreaks have occurred in Bangladesh (Ploquin et al., 2013) or India nearly every year (Islam et al., 2016). Recently in 2021, NiV outbreaks were recorded in the Kozhikode district of Kerala, a South Indian state (Yadav et al., 2022). Interpersonal transmission has been demonstrated in outbreaks in Bangladesh, therefore, therefore, NiV may have the potential to produce a deadly global pandemic (Gurley et al., 2007; Luby, 2013).

Nipah and henipaviral diseases are listed among the WHO research and development blueprint for priority diseases in emergency contexts (WHO, 2021). NiV and HeV bind to and enter host cells through their attachment (G) and fusion (F) glycoproteins. Their G glycoprotein recognizes and binds to the host cell surface receptors ephrin-B2 (EFNB2) and ephrin-B3 (EFNB3) (Bowden et al., 2008), which, in turn, triggers a conformational cascade reaction of F, leading to membrane fusion (Aguilar et al., 2010). Thus, the immunization strategy against HNVs mainly involves its G and F proteins. There are currently over 40 vaccines against HNVs in development (Gómez Román et al., 2022), including viral vector vaccines (Guillaume et al., 2004; Ploquin et al., 2013; Kalodimou et al., 2019; van Doremalen et al., 2019), subunit vaccines, virus-like particles (Liu et al., 2013), DNA vaccines, and mRNA vaccines. Currently, there are three NiV vaccines in clinical trials, a VSV-NiV-G vaccine (DeBuysscher et al., 2014; Prescott et al., 2015; DeBuysscher et al., 2016; Monath et al., 2022), a mRNA vaccine (Loomis et al., 2020; Loomis et al., 2021), and a HeV-sG subunit vaccine (Medicine, USNLo). The most advanced vaccine candidate is the subunit vaccine using soluble ectodomain of HeV G as the immunogen, which shown protection in a variety of animal models (Geisbert et al., 2021). Some research show that cellular immune response is important against henipaviruses (Pickering et al., 2016), indicating the need for safe and effective HNVs vaccines that can stimulate both cellular and humoral immune responses.

Immunogen design is critical to the success of a vaccine. The head domain of NiV G was found to be immunodominant and was the target of most of the serum neutralizing activity elicited by NiV G vaccination in rhesus macaques (Wang et al., 2022), while the soluble ectodomain of G was used in recombinant protein vaccine HeV-sG, and the full-length transmembrane G was used in most viral vector HNVs vaccines. In different vaccine candidates, the G protein is presented as disulfide-linked tetramer, transmembrane protein (Kalodimou et al., 2019; Monath et al., 2022), or monomer protein (Wang et al., 2022). It is valuable to use the same viral vector to present different forms of G protein for direct comparison, which will lead to a candidate vaccine that can stimulate the strongest immune response.

Since recombinant vaccines based on a replication-defective type 5 adenovirus vector (Ad5) platform can elicit rapid and efficient humoral and cellular immune responses and have been shown to be safe and effective against pathogens such as Ebola virus (Zhu et al., 2015; Zhu et al., 2017) and SARS-CoV-2 (Zhu et al., 2020a), the same platform was used in this study to construct candidate NiV vaccines delivering different forms of NiV G as the immunogen. The results showed that all three Ad5-NiV vaccine candidates efficiently and rapidly stimulate both humoral and cellular immune responses against HNVs in mice, with Ad5-NiVGe, which delivers the extracellular region of NiV-G, eliciting the strongest immune response. Furthermore, this research identified the potent conserved T cell epitope shared by NiV and HeV.

## 2 Materials and methods

### 2.1 Construction of Ad5-NiVG, Ad5-NiVGe and Ad5-NiVGs

The G glycoprotein gene from NiV-Bd (Gene ID: 920955) was used in the production of the recombinant adenovirus type-5 vector vaccine. Three Ad5-NiV vaccines delivering G glycoproteins of different lengths were constructed: Ad5-NiVG, expressing the full-length G glycoprotein; Ad5-NiVGe, expressing the soluble external domain of G protein; and Ad5-NiVGs, expressing the soluble head domain of G protein. The Ad5-NiV vaccines were produced as previously described (Zhu et al., 2015; Zhu et al., 2017). Briefly, the G-protein sequence of NiV was codon optimized and the tPA signal peptide was added to the sequence of NiV-Ge and NiV-Gs. The optimized G-protein sequences were synthesized (Applied Biological Materials Inc.) and cloned into pAdenoG vectors. For recombinant adenovirus packaging, the constructed plasmids were transfected into HEK293 cells using DNAfectin™ Plus Transfection Reagent (ABM, Canada) and purified *via* cesium chloride density gradient centrifugation. The obtained recombinant adenoviruses were tittered using the endpoint dilution method and subsequently tested for the presence of bacterial and mycoplasma contamination using the bacterial culture method and PCR, respectively (performed by Applied Biological Materials Inc.). Western Blot was performed according to the ProteinSimple (ProteinSimple, USA) user manual. Samples, i.e., cell precipitate and supernatant, primary antibody, i.e., mouse-specific anti-NiVGe protein serum diluted 1:500, 1:200 dilution of HRP-conjugated second antibodies, and chemiluminescent substrate were added to the designated wells of a 25-well microplate. After microplate loading, separation electrophoresis and immunodetection steps were performed in the capillary system (ProteinSimple, USA).

### 2.2 Protein expression and purification

The sequence of the extracellular region of G proteins from HeV and NiV (HNV-G) were codon-optimized, the tPA signal peptide and Strep-II tag were added at the N-terminus. Gene was synthesized (General Biosystems Co., Ltd.) and inserted into pcDNA3.1 (+).

The plasmid was transfected into Expi-293F cells for expression. After transfection, cells were placed on a shaker at 120 rpm and 37°C. After 72 hours of cell culture, the supernatant was centrifuged at 3,000 g for 15 min and filtered through a 0.45 μm filter (Thermo Fisher Scientific, USA). The protein was purified using the StrepTrap HP affinity column (GE Health Care, USA). Purified proteins were concentrated using 30 K ultrafiltration tubes (Merck Millipore, Germany) and permuted using PBS buffer at pH 7.4. Finally, the obtained proteins were characterized using SDS-PAGE and quantified with a BCA Protein Assay Kit (Thermo Fisher Scientific, USA).

### 2.3 Animal immunization

Specific pathogen-free (SPF) female mice (6-8 weeks) were used in this study for the vaccine immunization experiments. All animal experiments were reviewed and approved by the Animal Welfare and Ethics Committee (approval number: IACUC-SWGCYJS-2020-03, approval date December 2020), and the experimental animals were purchased from Beijing Viton Lever Laboratory Animal Technology Co.

Each mouse was immunized with a 1×10^7^ pfu adenovirus vector vaccine, while the protein subunit vaccine group was immunized with 10 µg of protein and 200 µg of aluminum adjuvant. As control, mice were injected with PBS. All mice were administered 100 µL using intramuscular injection.

### 2.4 ELISA

Purified NiV and HeV soluble G proteins were coated in 96-well ELISA plates using 50 mM carbonate buffer (pH=9.6) at a concentration of 1 μg/mL and incubated overnight at 4°C. After washing with PBST (PBS with 0.1% Tween20), all wells were blocked for 1 h at 37°C by adding 100 μL of PBS with 2% BSA. After the supernatant was discarded, the plate was washed three times with PBST. Subsequently, diluent (PBS with 0.2% BSA) and serum were added and serially diluted with two replicates per dilution, before incubation at 37°C for 1 h. The plate was washed three times with PBST after discarding the supernatant and adding a 1:50,000 dilution of HRP-conjugated goat anti-mouse IgG (Abcam, UK) at 100 μL/well. This was incubated for 45 min at 37°C. After discarding the supernatant, the plate was washed three times with PBST, and TMB solution (Solarbio Life Sciences, China) was added at 100 μL/well. ELISA Stop Solution (Solarbio Life Sciences, China) was then added at 50 μL/well after 5 minutes of color development, as the final enzyme marker. The absorbance was measured at 450 nm.

### 2.5 HIV backbone pseudovirus packaging

The HIV backbone pseudovirus was packaged as described previously (Li et al., 2020), and the optimized G-protein and F-protein sequences of NiV were constructed into pcDNA3.1 (+) to obtain the envelope protein plasmids pcDNA3.1-G and pcDNA3.1-F of the HIV backbone Nipah pseudovirus (synthesized by General Biologics Ltd.).A total of 4×10^6^ HEK-293T were preplaced in 10 cm cell culture dishes 24 h before transfection. Subsequently, cells were transfected with Lipofectamine 3000 transfection reagent (Invitrogen) and the pcDNA3.1-G and pcDNA3.1-F plasmids were transfected with the pNL4-3. Luc-R-E. The culture supernatant was collected and centrifuged at 3,500 g for 15 min, filtered through a 0.45 μm filter, and stored at -80°C after 60 h of transfection. Hendra pseudoviruses were packaged using the same method described above.

### 2.6 Pseudovirus neutralization experiment

Sera from each group were incubated at 56°C for 30 min. The inactivated serum was put into a 96-well cell culture plate for serial dilution with two replicates per dilution. A total of 50 μL/well of pseudovirus solution was added and then incubated for 1 h at 37°C. Thereafter, 50 μL/well of 293T cells (6×10^4^/well) were added and incubated for 48 h to express the luciferase. The culture supernatant was aspirated. The cells were then lysed by adding diluted cell lysis buffer (Promega, Luciferase Cell Culture Lysis 5X Reagent) and incubated at 800 rpm for 15 min. A total of 20 μL of aspirate of the lysed sample was placed into an opaque white 96-well plate and 100 μL of Luciferase Assay Reagent was added to each well. The fluorescence value was then assessed using a Promega Glomax fluorometer.

### 2.7 Luminex receptor competition inhibition assay

Microspheres were packed as described previously (Li et al., 2020). The isolated mouse serum was serial diluted using PBS (containing 1% BSA) with two replicates per dilution. They were then added to a black opaque 96-well plate. Magnetic beads coated with G_NiV-Bd_, G_NiV-My_, and G_HeV_ were added to the plate with 1,500 beads/well of each type. A total of 20 µL of diluted magnetic beads was added to each well. Next, 10 μL of diluted mouse ephrin-B2 Fc chimera biotinylated protein (R&D Systems, Minneapolis, USA) at a final concentration of 16.5 ng/mL was added to each well, before incubation at 800 rpm for 60 min. Subsequently, 10 µL of streptavidin-r-phycoerythrin (SAPE) at a concentration of 12 µg/mL was added to each well, before incubation at 800 rpm for 30 min. The supernatant was aspirated using a magnetic plate separator (Luminex) and washed three times with PBS (containing 1% BSA). The coated magnetic beads were then measured using a Luminex MAGPIX instrument (Luminex, USA).

### 2.8 Peptide pool synthesis

The NiV G protein has 602 amino acids in total. We selected the extracellular region of the G protein, i.e., from amino acids 72 to 602, for peptide library synthesis. We chose 15 amino acids for one peptide, with a step size of 4 and 11 overlaps for each peptide. Finally, we synthesized 128 peptides and desalted all peptides with 98% purity (92 (LQ-15) and 126 (IN-15) with purity >70%). The peptide library was synthesized by Gill Biochemistry, Shanghai, Co.

### 2.9 Cytometric bead array

The spleens of the mice were removed under aseptic conditions after euthanasia and ground. The mouse spleen cells were diluted to 4×10^6^/mL using 1640 medium containing 10% FBS, and 50 μL of the cell suspension was added to a 96-well cell culture plate. Thereafter, the peptide library or antigen diluent was added for stimulation. The 96-well plates were incubated in a cell culture incubator at 37°C for 3 days. After stimulation, the supernatant was centrifuged at 500 g for 5 min and collected. Cytokine secretions of the splenocytes were analyzed using mouse Th1/Th2/Th17 CBA Kit (BD Pharmingen, USA) following the previously described procedure. Supernatants were collected and aliquoted for the cytokine assay. Captured bead reagents were applied to the mixture of the 50 mL sample and PE-detection antibody. The reaction lasted 2 h in the dark at room temperature. All unbound antibodies were removed by the addition of 1 mL of wash buffer before centrifugation. Captured beads were then resuspended with 300 μL wash buffer and analyzed using FACS (BD). Six standard curves were acquired for each experiment using four-parameter linear fitting for the concentrations of serially diluted standards ranging from 0-5000 pg/mL.

### 2.10 ELISpot

The experimental method was the same as above, except that the cell suspension and the stimuli were added to a 96-well ELISpot plate (Mabtech, Sweden) coated with IFN-γ antibody, PMA and deionomycin (Dakewe Biotech Co., Ltd.) were added as positive controls, and the cells incubated without stimuli were used as negative controls. The ELISpot plates were incubated in a cell incubator for 36 h. The supernatant was discarded, the plates were washed 5 times with PBS, and the biotinylated antibody was added and incubated for 2 h at room temperature. The supernatant was discarded, the plate was washed 5 times with PBS, and the HRP secondary antibody was added and incubated at room temperature for 1 h. The supernatant was discarded, the plate was washed 5 times with PBS, TMB chromogenic solution was added for 6-8 min at 37°C, and the plate was washed with plenty of deionized water to stop the chromogenic reaction. After washing with deionized water, the plates were left to dry, and AT-Spot 3200 (Antai Yongxin Medical Technology, China) was used for the analysis.

### 2.11 Epitope prediction tools

Referring to the methods used for antigenic and epitope prediction in other studies, RANKPEP (http://imed.med.ucm.es/Tools/antigenic.pl) and ABCpred (https://webs.iiitd.edu.in/raghava/abcpred/) were selected for antigenic peptide prediction (Saha and Raghava, 2006; Saha and Raghava, 2007). The human leukocyte antigen (HLA) system and polymorphism frequency analyses were conducted using the Allele Frequency Net Database (http://www.allelefrequencies.net/hla6006a.asp) (Gonzalez-Galarza Faviel et al., 2019). The regions chosen were Australia, South Asia, Southeast Asia, and Sub-Saharan Africa. The alleles were selected for HLA-A∗02:01. T-cell epitopes of G_NiV-Bd_ were predicted using IEDB (SMM), NetCTL, PREDEP, and RANKPEP (**Table 1**).

**Table 1:** Programs used in this study for HLA-restricted cluster of differentiation (CD8) and T-cell epitope prediction.

Programs	URL
IEDB	http://tools.immuneepitope.org/main/
NetCTL	https://services.healthtech.dtu.dk/service.php?NetMHC-4.0
PREDEP	http://margalit.huji.ac.il/Teppred/mhc-bind/index.html

## 3 Results

### 3.1 Construction and characterization of Ad5-NiVG, Ad5-NiVGe, and Ad5-NiVGs

**Figure 1:**
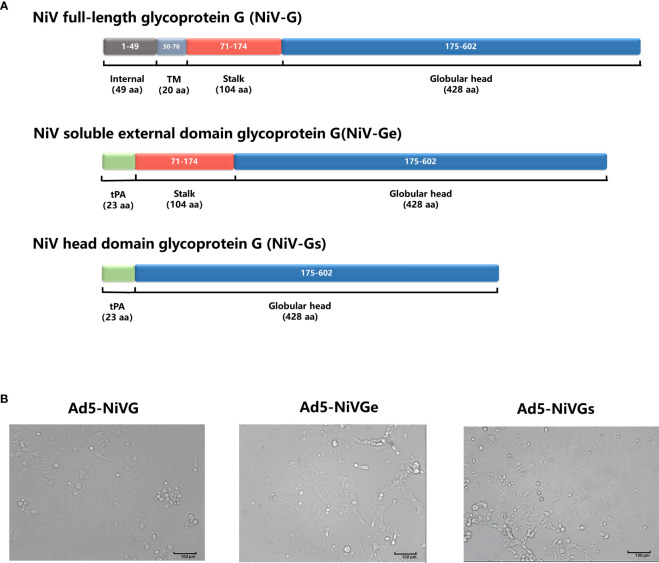
Construction and characterization of Ad5-NiVG, Ad5-NiVGe, and Ad5-NiVGs. **(A)** Schematic demonstration of NiV full-length glycoprotein G (NiV-G), NiV-soluble external domain glycoprotein G (NiV-Ge), and NiV head domain glycoprotein G (NiV-Gs) carried by adenovirus. Colored rectangles represent individual protein subdomains, and bracketed text denotes the start and end of the amino acid sequences of each domain. **(B)** cytopathology formed by recombinant adenovirus infection in 293T cells.

To investigate the optimal performance of NiV G protein as a vaccine antigen, three recombinant adenovirus type 5 vector-based NiV vaccines (Ad5-NiV) delivering different forms of NiV G were constructed (**Figure 1A**). After infection of 293T cells with three recombinant adenoviruses, the cellular appearance was characterized as a grape bead-like lesion (**Figure 1B**). Western blot identification results show that the protein blots were detected only in the cell lysate after Ad5-NiVG infection and only in the supernatant after Ad5-NiVGs infection, while in both cell lysate and supernatant after Ad5-NiVGe infection (**Figure S1**).

### 3.2 Ad5-NiV vaccines induced robust HNVs G-specific antibody responses

**Figure 2:**
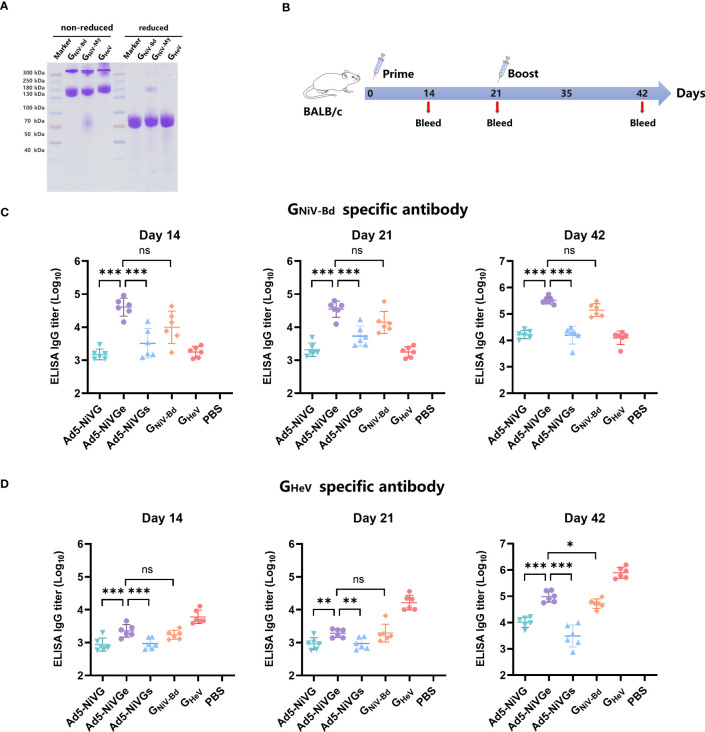
HNVs G-specific antibody response induced by NiV vaccines. **(A)** G_NiV-My_ (G protein of Malaysia Nipah virus), G_NiV-Bd_ (G protein of Bangladesh Nipah virus), and G_HeV_ (G protein of Hendra virus) were expressed in the Expi293F suspension cells and purified using affinity chromatography. Purified proteins were assessed using reduced SDS-PAGE gel. **(B)** Vaccination experimental design. Female, 6–8-week-old BALB/c mice (6 per group) were used for two intramuscular immunizations on days 0 and 21. The Ad5-NiV group was immunized with doses of 10^7^ plaque-forming units (PFU), the protein subunit vaccine group was immunized with 10 µg of protein and 200 µg of aluminum adjuvant, and the control group was injected with PBS. **(C)** Specific antibody titers against G_NiV-G-Bd_ on days 14, 21, and 42 postvaccination. **(D)** Specific antibody titers against G_HeV-G_ on days 14, 21, and 42 postvaccination. The data are presented as the mean log10 IC50 titer ± SEM. Error bars represent the standard deviation from the mean of six biological replicates. Student’s *t* test was performed for all comparisons, and a p-value < 0.05 was considered statistically significant. *0.01< p-value ≤ 0.05, **0.001< p-value ≤ 0.01, ***p-value ≤ 0.001. N.S. indicates no significant statistical difference (p> 0.05).

Recombinant proteins of G_NiV-Bd_, G_NiV-My_ and G_HeV_ were prepared, and consistent with reported studies (Maar et al., 2012; Ortega et al., 2022), they formed mainly dimers and tetramers when separated on non-reducing SDS-PAGE gel, whereas were monomeric in reducing SDS-PAGE gel (**Figure 2A**). G_NiV-Bd_ and G_HeV_ were used for detecting specific antibody *via* ELISA. G_NiV-Bd_, G_NiV-My_, and G_HeV_ were used to detect neutralizing antibody responses *via* the Luminex assay.

In order to test the immunogenicity of Ad5-NiV vaccines, BALB/c mice were immunized intramuscularly with 1 × 10^7^ PFU of Ad5-NiV vaccines or recombinant protein vaccines as control on day 0 and day 21 (**Figure 2B**).

The HNVs G-specific antibody responses elicited by these vaccines were detected by enzyme-linked immunosorbent assay (ELISA) (**Figures 2C, D**). First, the ELISA results revealed that G_NiV-Bd_ specific antibody levels induced by Ad5-NiVGe were significantly higher than those induced by the Ad5-NiVGs and Ad5-NiVG groups. Moreover, they all were significantly higher than the levels observed in the G_NiV-Bd_ protein vaccine group (**Figure 2C**). The mean log10 antibody titer of the Ad5-NiVGe group in the ELISA against G_HeV_ 14 days after immunization was 3.35 (**Figure 2D**), which was not significantly different from that of the G_NiV-Bd_ subunit vaccine group. The robust HNVs G-specific antibody response was stimulated after booster immunization, and the antibody titer of the vaccinated mice was generally increased. The level of G_NiV-Bd_-specific antibodies in Ad5-NiVGe vaccinated mice increased 7.9-fold, with a mean log10 titer of 5.51 (**Figure 2C**). The mean titer of G_NiV-Bd_-specific antibodies in the Ad5-Ge group was 2.3-fold higher than that of the G_NiV-Bd_ vaccine group (**Figure 2C**). The cross-reactive antibodies stimulated by Ad5-NiVGe increased 8.1-fold, which was significantly higher than the G_NiV-Bd_ vaccine group (**Figure 2D**). All three Ad5-NiV vaccines rapidly elicited potent specific and cross-reactive antibody responses against HNVs, and the antibody titers were significant higher in the Ad5-NiVGe vaccine group than in the Ad5-NiVGs and Ad5-NiVG groups.

### 3.3 Ad5-NiV vaccines rapidly elicited neutralizing antibodies that block the receptor binding

**Figure 3:**
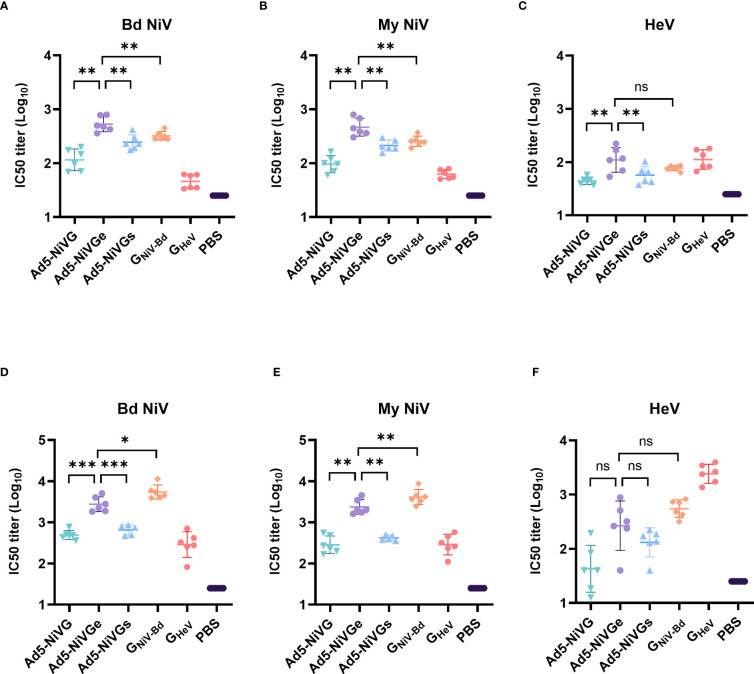
NiV vaccines elicit neutralizing antibodies that block receptor binding. Luminex receptor competition inhibition assay on day 14 for **(A)** G_NiV-Bd_, **(B)** G_NiV-My_, and **(C)** G_HeV_. Luminex receptor competition inhibition assay on day 42 for **(D)** G_NiV-Bd_, **(E)** G_NiV-My_, and **(F)** G_HeV_. The data are presented as the mean log10 IC50 titer ± SEM. Error bars represent the standard deviation from the mean of six biological replicates. Student’s *t* test was performed for all comparisons, and a p-value < 0.05 was considered statistically significant. *0.01< p-value ≤ 0.05, **0.001< p-value ≤ 0.01, ***p-value ≤ 0.001. N.S. indicates no significant statistical difference (p> 0.05).

To evaluate the neutralizing antibody responses stimulated by Ad5-NiV vaccines, sera were collected on days 14 and 42 postvaccination. Neutralizing antibodies against HeV and NiV were detected by Luminex receptor competition inhibition assay (**Figure 3**). In the ephrinB2 inhibition assay against G_NiV-Bd_ and G_NiV-My_ on day 14 postimmunization, the IC50 titer of the Ad5-NiVGe group was significantly higher than those of the Ad5-NiVGs and Ad5-NiVG groups, and significantly higher than that of the G_NiV-Bd_ group (**Figures 3A, B**). In the ephrinB2 inhibition assay against G_HeV_, the titer of the Ad5-NiVGe group was significantly higher than those of the Ad5-NiVGs and Ad5-NiVG groups, while no significant difference was observed in the IC50 titers of the G_NiV-Bd_ groups on day 14 (**Figure 3C**).

The specific neutralizing and cross-neutralizing antibody titers of the vaccines increased significantly after booster immunization (**Figures 3D-F**). The Ad5-NiVGe group elicited higher levels of neutralizing antibodies against G_NiV-Bd_ than the Ad5-NiVGs and Ad5-NiVG groups (**Figure 3D**). The Ad5-NiVGe group showed a 2.5-fold increase in cross-neutralizing antibody levels against G_HeV_, which was not significantly different from that of the G_NiV-Bd_ vaccine group (**Figure 3F**).

### 3.4 Ad5-NiV vaccines elicited neutralizing antibodies that potently neutralize HNVs pseudoviruses

**Figure 4:**
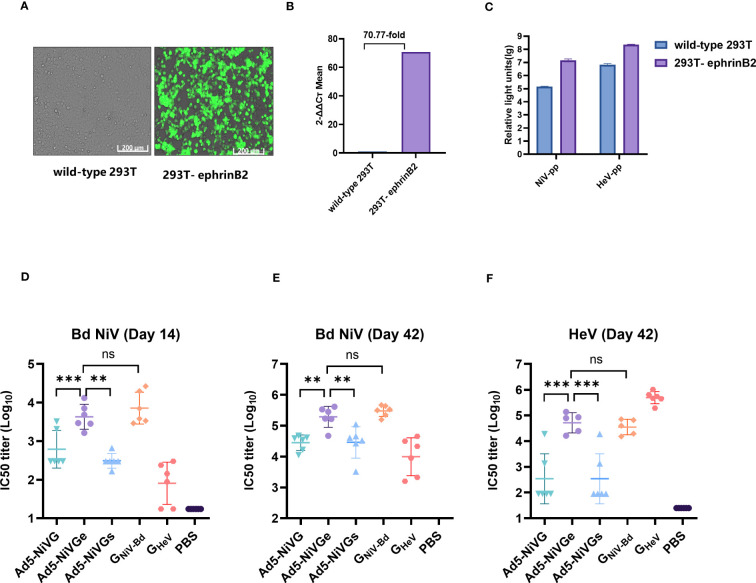
Neutralizing activity of serum detected by pseudoviruses. **(A)** Cell images of 293T-ephrinB2 cells’ expression of eGFP gene. **(B)** EFNB2 gene expression rate (%) compared with the wild -type 293T cell line. **(C)** Generation of the HNV pseudoviruses. 293T-ephrinB2 and wild-type 293T cells were infected with HIV backbone pseudoviruses. **(D)** Pseudovirus neutralization assay on day 14 with NiV-PP. Pseudovirus neutralization assay on day 42 with NiV-PP **(E)** and HeV-PP **(F)**. The data are presented as the mean log10 IC50 titer ± SEM. Error bars represent the standard deviation from the mean of six biological replicates. Student’s *t* test was performed for all comparisons, and a p-value < 0.05 was considered statistically significant. *0.01< p-value ≤ 0.05, **0.001< p-value ≤ 0.01, ***p-value ≤ 0.001. N.S. indicates no significant statistical difference (p> 0.05).

The HIV backbone pseudoviruses were packaged as described previously, and the optimized NiV G and F protein sequences were constructed into pcDNA3.1 (+) to obtain the envelope protein plasmids pcDNA3.1-G and pcDNA3.1-F of the HIV backbone Nipah pseudovirus. Studies have shown that ephrinB2 is a functional receptor of NiV and HeV, so an ephrinB2 overexpression 293T cell line was successfully constructed (**Figure 4A**). As shown in the RT-PCR analysis, compared with wild-type 293T cell line, the expression rate of the 293T-ephrinB2 cell line was about 7077% (**Figure 4B**). The results show that the titer of 293T-ephrinB2 cells was significantly higher than that of wild-type 293T cells when the cells were infected with HIV backbone pseudovirus (**Figure 4C**). The construction of stable transfected cells improved the efficiency of pseudovirus preparation and contributed to the reliability and stability of the assay. Therefore, HIV backbone pseudovirus infection of 293T-ephrinB2 cells was used to assess the level of neutralizing antibodies in serum.

The results of the pseudovirus neutralization assay were consistent with the Luminex receptor competition inhibition assay (**Figures 4D–F**). The pseudovirus neutralization assay showed that the Ad5-NiVGe group had significantly higher levels of neutralizing antibodies than Ad5-NiVG and Ad5-NiVGs groups on day 14 postimmunization (**Figure 4D**). Moreover, similarly high levels of neutralizing antibody responses against HeV were maintained for 21 days after booster immunization. (**Figure 4F**). The data above suggest that Ad5-NiVGe was able to induce the highest nAbs among the Ad5-NiV vaccines.

### 3.5 Ad5-NiV vaccines triggered specific cellular immune responses in mice

**Figure 5:**
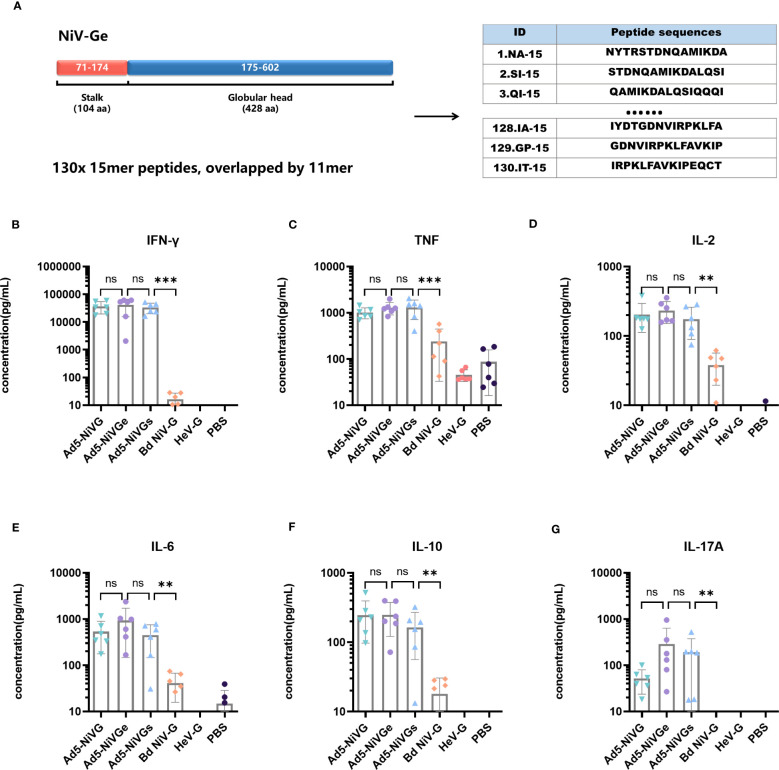
Specific cellular immune response elicited by Ad5-NiV vaccines. BALB/c mice (n = 6 per group) were immunized intramuscularly with a dose of 10^7^ PFU of Ad5-NiV once on day 0, or twice, on days 0 and 21. The murine splenocytes were collected and single-cell suspensions were prepared on day 14 and day 35, stimulated with NiV-G overlapping pools or G_HeV_, and analyzed by cytometric bead array. **(A)** Schematic overview of the strategies used for peptide mapping. The peptide pool was synthesized and contained the sequence of the G-protein external domain, i.e., starting from the first amino acid at the N-terminal to the C-terminus (amino acids 71 - 602). There were 15 amino acids for one peptide, with a step size of 4 and 11 overlaps for each peptide. Cytometric bead array measuring cytokine concentrations, i.e., IFN-γ **(B)**, TNF **(C)**, IL-2 **(D)**, IL-6 **(E)**, IL-10 **(F)**, and IL-17A **(G)**, in the supernatant of NiV-Ge peptide-pool-stimulated 14-day immunized mouse splenocytes. The data are presented as the mean log10 IC50 titer ± SEM. Error bars represent the standard deviation from the mean of six biological replicates. Student’s *t* test was performed for all comparisons, and a p-value < 0.05 was considered statistically significant. **0.001< p-value ≤ 0.01, ***p-value ≤ 0.001. N.S. indicates no significant statistical difference (p> 0.05).

In order to evaluate the cellular immune response initiated by the Ad5-NiV vaccine, a peptide pool was synthesized which contained the sequence of the external domain of G protein such as starting from the first amino acid at the N-terminal to the C-terminus (amino acids 71 - 602) (**Figure 5A**). The peptide pool contained 130 peptides, each with 15 amino acids and a step size of 4mer, i.e., overlapped by 11mer (**Figure 5A**).

Cytokines secreted by Ad5-NiV vaccines were detected using a Cytometric Bead Array kit. Among the six cytokines tested, IL-2, IFN-γ, and TNF are associated with the Th1-type cellular immune response, IL-6 and IL-10 with the Th2-type response, and IL-17A with the Th17-type response.

Cytokine levels in the secretory supernatant of splenocytes from peptide-pool-stimulated candidate Ad5-NiV-vaccinated mice were significantly higher than in the subunit vaccine group (**Figures 5B-G**). There was no significant difference in the levels of cytokines stimulated by the three Ad5-NiV vaccines, with IFN-γ concentrations reaching 30,000 pg/mL (**Figure 5B**) and TNF concentrations reaching 1,000 pg/mL (**Figure 5C**). Cross-cellular immune responses against HeV were detected among vaccinated mice using G_HeV_ as the stimulus (**Figure S2**). The Ad5-NiVG vaccines stimulated significantly higher cytokine levels than the subunit vaccines, and the three adenoviral vector vaccines stimulated IFN-γ concentrations ranging from 200 pg/mL to 900 pg/mL (**Figure S2A**), with mean TNF concentrations above 150 pg/mL (**Figure S2B**). Ad5 vectors without antigenic genes fail to stimulate effective cellular immune responses in mice (**Figure S6**).

Cellular immune responses induced by the Ad5-NiV vaccines were tested again 14 days after booster immunization (**Figure S3**). The results showed that the Ad5-NiV vaccines stimulated a Th1-dominant immune response. The three Ad5-NiV vaccines induced significantly higher cytokine levels than the PBS group, but there was no significant difference between the vaccine groups. The results of cytokine assays using G_HeV_ as a stimulus showed that the Ad5-NiV vaccine stimulated a cross-cellular immune response (**Figure S4**).

### 3.6 The potent H-2d-Restricted CD8+ T cell epitope peptide DTLYFPAVGFL shared by NiV and HeV were identified

**Figure 6:**
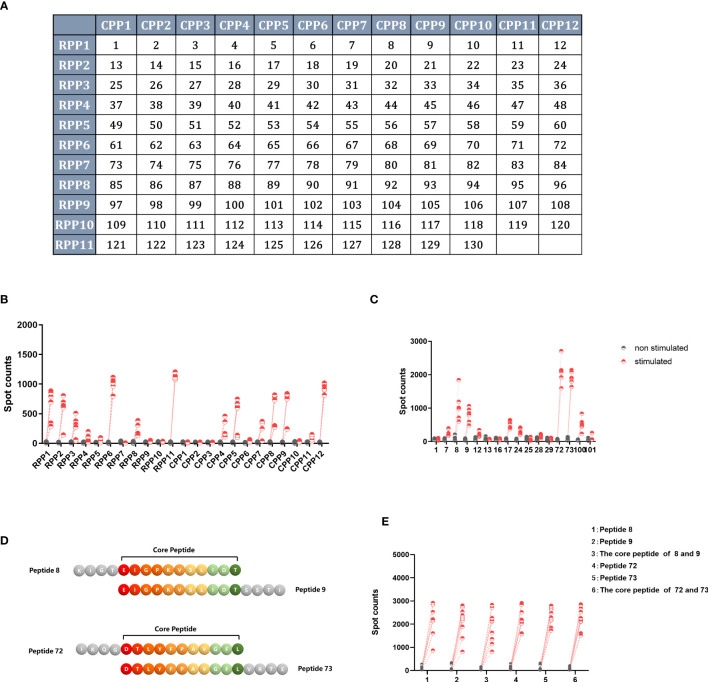
Screening for H2-b-restricted T-cell epitopes of NiV-G protein. BALB/c mice (n = 3 per group) were immunized once intramuscularly with a dose of 10^7^ PFU of Ad5-NiVGe. Mice inoculated with PBS were used as controls. Splenocytes were collected and single-cell suspensions were prepared on day 14 postimmunization and stimulated with pools of 11 overlapping 15mer peptides of NiV-G analyzed using the IFN-γ ELISpot assay. **(A)** The two-dimensional peptide matrix system. All 130 peptides (P1 to P130) tested were organized into a two-dimensional grid: columns and rows designate the peptides that constitute the corresponding random and overlapping peptide pools, respectively. Horizontal rows (RPP)1–(RPP)12 and vertical columns (CPP)1–(CPP)11 contain 10–12 peptides, and each cell of the matrix represents an individual 15mer peptide. In this manner, testing of the 130 individual peptides was replaced with testing of the 23 peptide pools. **(B)** IFN-γ^+^ responses of Ad5-NiVGe-immunized mouse splenocytes stimulated with the 23 peptide pools. **(C)** Screening for positive individual peptides. Peptides that tested positive were split into 16 individual peptides for the IFN-γ ELISPOT assay. **(D)** Display of overlapping amino acids. **(E)** IFN-γ secretion levels stimulated by core peptides of positive peptides. Three biological replicates for each experimental group.

A total of 130 peptides were screened twice using a two-dimensional peptide matrix system using ELISpot, and mice with splenocytes removed 14 days after single immunization with Ad5-NiVGe were used to assess the ability of the peptide pools to induce IFN-γ secretion. In the first experiment, 130 peptides were subgrouped into 23 peptide pools, and it was observed that some combinations of peptides were more effective in stimulating IFN-γ secretion in vaccinated mouse splenocytes (**Figures 6A, B**).

Subsequently, we reconstituted these more effective peptide combinations into 14 new peptide pools using the two-dimensional peptide matrix system, observing that seven of the newly composed peptide pools produced significantly higher levels of IFN-γ than the background (**Figure S5**). The seven newly composed peptide pools were peptide pools 1, 2, 5, 7, 11, and 14. The most effective peptide library was peptide 11, which only contained peptides 72 and 73 and stimulated the highest level of IFN-γ production, with an average spot count of 1000 (**Figure S5B**).

The single peptide with the best effect was stimulated separately to obtain the final screening result (**Figure 6C**). In agreement with the previous results, peptides 72 (IKQGDTLYFPAVGFL) and 73 (DTLYFPAVGFLVRTE) produced the highest levels of IFN-γ, followed by peptides 8 (KIGTEIGPKVSLIDT) and 9 (EIGPKVSLIDTSSTI). The core peptide sequence (EIGPKVSLIDT) of peptides 8 and 9 is located in the stem region of G_NiV-Bd_. The core peptide sequence (DTLYFPAVGFL) of peptides 72 and 73 is that of amino acids 360-370 of G_NiV-Bd_, which is located in the head region of G_NiV-Bd_ (**Figure 6D**). In addition, to determine whether their core sequences would produce the same efficacy, we used both the four individual peptides and their core peptides as stimulators to detect the secretion level of IFN-γ, and the results showed that their core peptides showed the same stimulatory effect as the individual peptides (**Figure 6E**).

### 3.7 Multi-method prediction suggests that the peptide DTLYFPAVGFL might be an HLA-A0201-restricted CD8+ T cell epitope in humans

RANKPEP was used to predict the antigenic determinants on G protein (Kolaskar and Tongaonkar, 1990). Moreover, ABCpred (Saha and Raghava, 2006), using feed-forward (FNN) and recurrent neural network (RNN) linear epitope prediction, was also utilized. The core peptide sequence (DTLYFPAVGFL) of peptides 72 and 73 scored high on the prediction site.

A previous study concluded that predicted peptide-MHC binding is the main basis for predicting T-cell epitopes (Sanchez-Trincado et al., 2017). The regions chosen were Australia, South Asia, Southeast Asia, and Sub-Saharan Africa. G_NiV-Bd_ T-cell epitopes were predicted using IEDB (Tepitool) (Paul et al., 2016), NetCTL (Andreatta and Nielsen, 2016) and PREDEP,which take into account multiple features of the protein (Desai and Kulkarni-Kale, 2014). The results from the prediction procedure show that peptide DTLYFPAVGFL exhibits a high binding affinity with class I alleles. The epitope DTLYFPAVGFL predicted a score and percentile ranking of 0.0018 and 11%, respectively (IEDB), and the affinity with the allele HLA-A0201 was 3188.28 nM and the rank was 7.5% (NetCTL). Since in PREDEP prediction of MHC allele and peptide length can only choose 9 or 10, the result exhibits the peptide TLYFPAVGFL energy score of -5.80 and ranking as 14.

The epitope DTLYFPAVGFL is conserved in NiV and HeV. Experimental and multimethod predictions suggested that the peptide DTLYFPAVGFL was a H-2d-restricted CD8+ T-cell epitope in mice and most likely also as an HLA-A0201-restricted CD8+ T cell epitope in humans, which is valuable in human clinical trials and the study of epitope vaccines.

## 4 Discussion

HNVs are regarded as emerging pathogens with notable epidemic potential. The unpredictability of NiV outbreaks, the high fatality rate, and the paucity of specific medical countermeasures to control infections in humans suggest an urgent need to prepare for future outbreaks.

A safe, efficacious vaccine would be a powerful means of preventing infection and the transmission of the virus. More than 40 NiV vaccines have been tested in various animal models, including virus vectored vaccines, recombinant protein subunit vaccine, nucleic acid vaccine, and virus-like particle (VLP) vaccines. The recombinant soluble G (sG) HeV subunit vaccine, Equivac^®^ HeV has completed clinical trials and has been verified to provide protection in cats, ferrets, horses, and AGMs (McEachern et al., 2008; Pallister et al., 2013; Middleton et al., 2014; Geisbert et al., 2021).

Ad5 (E1-, E3-) vector vaccines are promising candidates because of their established safety profiles and ease of genetic modification. Vaccine candidates prepared using the Ad5 (E1-, E3-) vector against a variety of viruses have high abilities in eliciting rapid and effective both cellular and humoral immune responses (Zhu et al., 2015; Zhu et al., 2017; Zhu et al., 2020a; Zhu et al., 2020b). Consistent with other paramyxoviruses, the immunization strategies for NiV and HeV mainly focus on G and F proteins, to which nearly all neutralizing antibodies are directed (Amaya and Broder, 2020). Two genetically divergent strains of NiV have been included in different clinical and epidemiological findings, i.e., NiV-My and NiV-Bd. NiV-Bd is considered to have a higher fatality rate of almost 75%, as compared to the rate of 40% related to NiV-My. And NiV-Bd was the currently circulating strain (Soman Pillai et al., 2020; Johnson et al., 2021), thus G_NiV-Bd_ was chosen as the antigen for the Ad5-NiV vaccine construction in this study.

In this research, three antigen forms with different lengths of NiV G were delivered by Ad5 vector respectively and their immunogenicity were evaluated in BALB/c mice. The results showed that all three Ad5-NiV vaccines elicited specific antibody responses against G_NiV-Bd_ post initial and boost immunization. Antibody levels induced by Ad5-NiVGe were significantly higher than those in the other Ad5-vectored and recombinant protein groups at day 14 suggesting that Ad5-NiVGe can stimulate antibody responses more rapidly. A more robust HNVs G-specific antibody response was stimulated after booster immunization. In the ephrinB2 inhibition assay and pseudoviruse neutralizing assay, the Ad5-NiV vaccines were shown to elicit potent neutralizing antibody responses against both NiV and HeV.

The Ad5-NiVGe vaccine induced a stronger humoral immune response than Ad5-NiVG or Ad5-NiVGs. As shown in Western Blot analysis, NiV-Ge was detected in both cell lysate and supernatant as dimers and tetramers, while NiV-G was detected only in lysate as monomers and dimers, and NiV-Gs was detected only in supernatant as monomers. The results implied that the full-length G protein expressed by the Ad5-NiVG were stably present on the cell surface through its transmembrane domain, while the Ge and Gs expressed by the Ad5-NiVGe and Ad5-NiVGs, respectively, were secreted outside the cell. In addition, the stalked domain of NiV-Ge may help to maintain its stability by oligomerization. It was found that the cysteines in the stalk of NiV G protein assist in maintaining oligomeric stability by participating in inter-subunit disulfide bond formation (Xu et al., 2008). Thus, Ad5-NiVGe may be an effective vaccine candidate against HNVs through a combination of high expression and stability of the target antigen.

Cytokine levels in the secretory supernatant of splenocytes from the G_NiV-BD_ peptide pool and the G_HeV_-stimulated candidate Ad5-NiV-vaccinated mice at day 14 were significantly higher than in the recombinant protein vaccine group. There was no significant difference in the levels of cytokines stimulated by the three Ad5-NiV vaccines. After booster immunization, the protein vaccine group still failed to stimulate an effective cellular immune response.

Ad5-NiV vaccines require no adjuvants and are clearly efficacious as a single immunization strategy. In addition, they are able to induce both cell-mediated and humoral immune responses. This may contribute to the extensive tissue tropism of Ads and their ability to drive strong expression of the target antigen, which can induce an innate immune response parallel to vaccine antigen expression. Activation of innate responses appears to involve several pathways, including at least two toll-like receptors, i.e., toll-like receptor 2 and toll-like receptor 9, and the type I interferon (Lasaro and Ertl, 2009; Mendonça et al., 2021).

The amino acid homology of the G proteins of Hendra virus and Nipah virus is about 79%. Developing a universal vaccine against these two deadly pathogens can significantly reduce the cost and improve the accessibility of the vaccine. The Ad5-NiVGe vaccine elicited the strongest antibody response against the G_HeV_. However, similar to recombinant protein vaccine, the level of cross-neutralizing antibody response against the G_HeV_ was limited. Further modification of antigen is needed to enhance the broad-spectrum protection of the vaccine.

A number of studies have been conducted to design epitope-based NiV vaccines by utilizing in silico approaches (Parvege et al., 2016; Mohammed et al., 2020; Soltan et al., 2021), while studies have confirmed the identification of potential H2-b-restricted NiV-G CD8 and CD4 T-cell peptide epitopes (Kalodimou et al., 2019), other conserved T-cell epitopes targeting both NiV and HeV remain unidentified. Initially, we screened for G_NiV-Bd_ epitopes by overlapping peptide pools using a two-dimensional peptide matrix system. The most effective peptides 72 and 73 located in the head region of G_NiV-Bd_ stimulated the highest level of IFN-γ production, The core peptide sequence (DTLYFPAVGFL) of peptides 72 and 73 is conserved in the NiV and HeV. The peptide DTLYFPAVGFL obtained high prediction scores in the antigenic peptide prediction and prediction sites with an affinity for human MHC-I molecules. This implies that the DTLYFPAVGFL epitope may provide valid information on the mechanism of HNVs-specific cellular immunity and the epitope-based vaccine design.

In conclusion, the constructed Ad5-NiVGe vaccine in this study could efficiently and rapidly stimulate humoral and cellular immune responses against both NiV and HeV, further experiments are required to verify the protective effect of this vaccine in different animal models.

## Data availability statement

The original contributions presented in the study are included in the article/**Supplementary Material**. Further inquiries can be directed to the corresponding authors.

## Author contributions

All authors contributed to the article and approved the submitted version. JZ and JX supervision and conceptualization the research. XH and YL contributed to the experimental research and methodology of this study. RL, ShaW and LY contribute to experimental research. ShuW, YY and XZ contribute to the methodology and manuscript revision. XH and YL drafted and corrected the manuscript, with input from all the authors.
