# Supplementary material for: Nipah virus attachment glycoprotein ectodomain delivered by type 5 adenovirus vector elicits broad immune response against NiV and HeV
Source: Front Cell Infect Microbiol. 2023 Jul 27;13:1180344. doi: 10.3389/fcimb.2023.1180344 (PMC10413271; doi:10.3389/fcimb.2023.1180344)
Supplement: Supplementary file 1 [file DataSheet_1.pdf]

Supplementary Materials

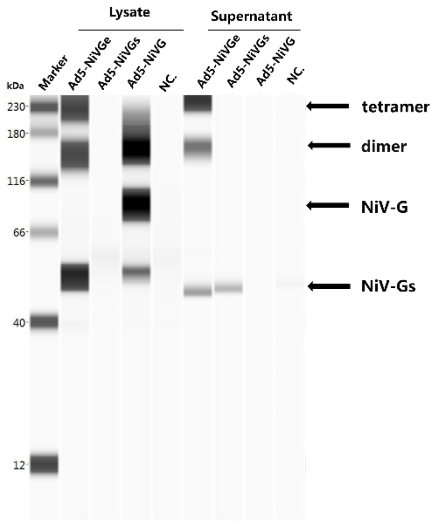

Figure S1. Western blot identification of recombinant Nipah virus glycoprotein produced by HeK-293T cells infected with Ad5-NiV. 293T cells were infected with 1×10^6pfu Ad5-NiVG, Ad5-NiVGe and Ad5-NiVGs, respectively. Lysates and culture supernatants were collected from cell cultures infected with Ad5-NiV or uninfected controls (NC.) after 48 h of infection. The mouse-specific anti-NiVGe protein serum was used as the primary antibody to characterize the antigen by WB. The black solid arrows represent NiV-G. In non-reducing conditions, monomer and dimer were detected only in lysates after Ad5-NiVG infection of cells, while the monomer was detected only in supernatants after Ad5-NiVGs infection. After Ad5-NiVGe infection, dimers and tetramers were detected in both cell lysates and supernatants. The WB identification experiments were performed in three biological replicates, while one of the representative results is shown.

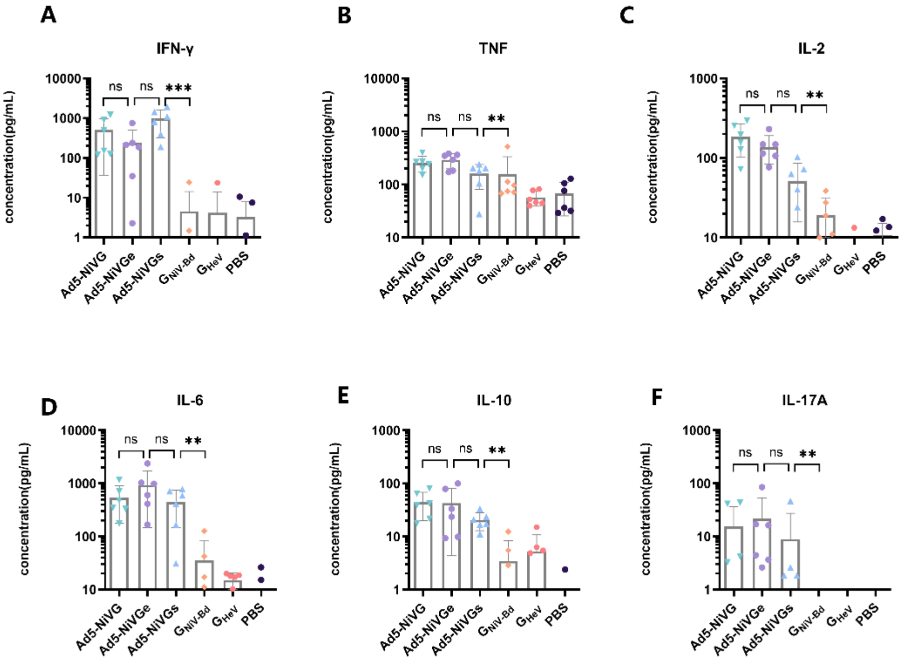

Figure S2. Cellular immune response against HeV elicited by Ad5-NiV vaccines after 14 days of primary immunization. Cytometric bead array measuring for IFN-γ(a), TNF(b), IL-2(c), IL-6(d), IL-10(e) and IL-17A(f) cytokine concentrations in the supernatant of G_{HeV} stimulated immunized 14-day mouse splenocytes. The data are presented as the mean log10 IC50 titer ±SEM. Error bars represent the standard deviation from the mean of six biological replicates. Student's t test was performed for all comparisons, and a P-value < 0.05 was considered statistically significant; * 0.01< P-value ≤ 0.05; ** 0.001< P-value ≤ 0.01; *** P-value ≤ 0.001;

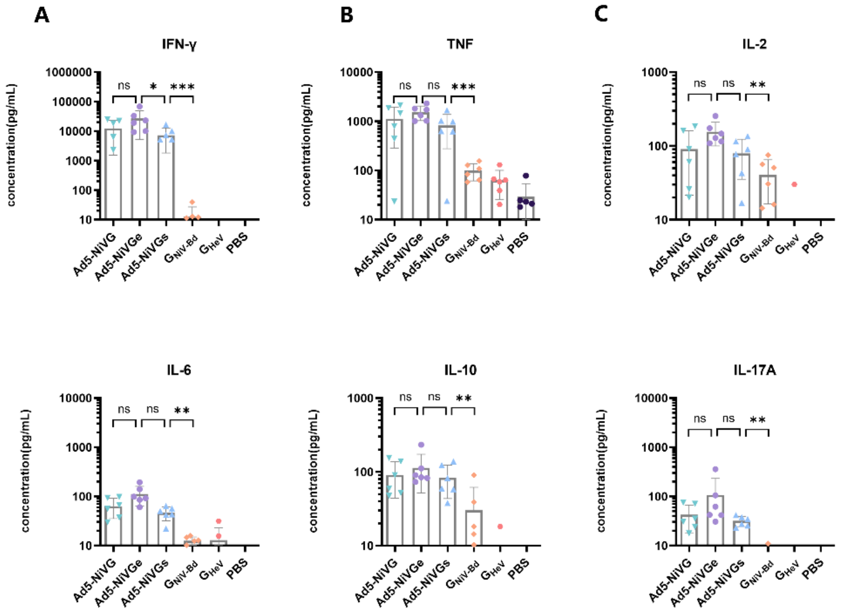

Figure S3. The specific cellular immune response elicited by Ad5-NiV vaccines after 14 days of booster immunization. Cytometric bead array measuring for IFN-

γ(a), TNF(b), IL-2(c), IL-6(d), IL-10(e) and IL-17A(f) cytokine concentrations in the supernatant of G_{HeV} stimulated immunized 14-day mouse splenocytes. The data are presented as the mean log10 IC50 titer ±SEM. Error bars represent the standard deviation from the mean of six biological replicates. Student's t test was performed for all comparisons, and a P-value < 0.05 was considered statistically significant; * 0.01< P-value ≤ 0.05; ** 0.001< P-value ≤ 0.01; *** P-value ≤ 0.001;

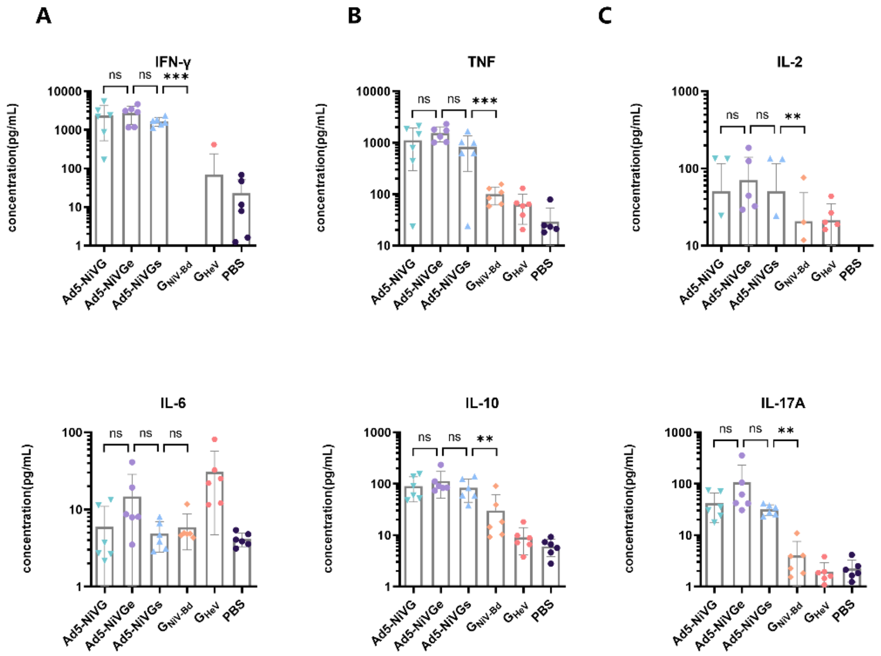

Figure S4. Cellular immune response against HeV elicited by Ad5-NiV vaccines after 14 days of booster immunization. Cytometric bead array measuring for IFN-γ(a), TNF(b), IL-2(c), IL-6(d), IL-10(e) and IL-17A(f) cytokine concentrations in the supernatant of G_{HeV} stimulated immunized 14-day mouse splenocytes. The data are presented as the mean log10 IC50 titer ±SEM. Error bars represent the standard deviation from the mean of six biological replicates. Student's t-test was performed for all comparisons, and a P-value < 0.05 was considered statistically significant; * 0.01< P-value ≤ 0.05; ** 0.001< P-value ≤ 0.01; *** P-value ≤ 0.001;

A

	1	2	3	4
5	1	16&17	31;32;33	48
6	4;5	19;20;21	36;37	67
7	7;8.9	24;25	40;41	79
8	12;13	28;29	43;44;45	

9	10	11	12	13	14
61	68;69	72;73	80;81	84	100;101

B

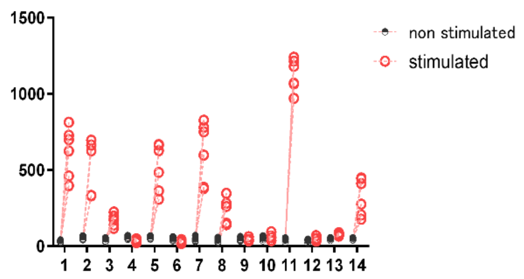

Figure S5. Screening for H2-b-restricted T cell epitopes of NiV-G protein. (a)Second two-dimensional peptide matrix system. Reconstituted tested positive

peptide combinations into 14 new peptide pools for IFN-γ ELISPOT assay. (b)Screening for positive individual peptides. Three biological replicates for each experimental group.

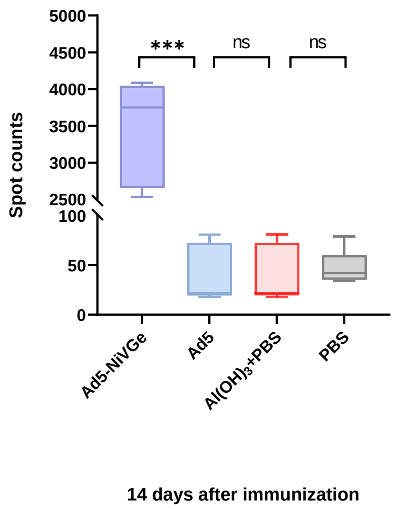

Figure S6. Detection of Cytokine-Secreting Cells by Enzyme-Linked Immunospot. Mice were immunized with Ad5-NiVGe and Ad5 vectors without the antigenic gene, respectively, while $Al(OH)_3$ and PBS groups were added as controls, and the level of IFN-γ secretion by splenocytes was measured using a peptide pool of NiV-G as a stimulus 14 days after administration. Error bars represent the standard deviation from the mean of three biological replicates. Student's t-test was performed for all comparisons, and a P-value < 0.05 was considered statistically significant; * 0.01< P-value ≤ 0.05; ** 0.001< P-value ≤ 0.01; *** P-value ≤ 0.001;
